# Leptin Receptor Deficiency Results in Hyperphagia and Increased Fatty Acid Mobilization during Fasting in Rainbow Trout (*Oncorhynchus mykiss*)

**DOI:** 10.3390/biom12040516

**Published:** 2022-03-29

**Authors:** Jamie L. Mankiewicz, Matthew J. Picklo, Joseph Idso, Beth M. Cleveland

**Affiliations:** 1 National Center for Cool and Cold Water Aquaculture, USDA/ARS, Kearneysville, WV 25430, USA; jamie.mankiewicz@usda.gov; 2 Human Nutrition Research Center, USDA/ARS, 2420 2nd Ave. North, Grand Forks, ND 58203, USA; matthew.picklo@usda.gov (M.J.P.); joseph.idso@usda.gov (J.I.)

**Keywords:** leptin receptor, CRISPR, gene editing, growth, rainbow trout, fatty acid, feeding, fasting, POMC

## Abstract

Leptin is a pleiotropic hormone known for regulating appetite and metabolism. To characterize the role of leptin signaling in rainbow trout, we used CRISPR/Cas9 genome editing to disrupt the leptin receptor (LepR) genes, *lepra1* and *lepra2*. We compared wildtype (WT) and mutant fish that were either fed to satiation or feed deprived for six weeks. The LepR mutants exhibited a hyperphagic phenotype, which led to heavier body weight, faster specific growth rate, increased viscero- and hepatosomatic indices, and greater condition factor. Muscle glycogen, plasma leptin, and leptin transcripts (*lepa1*) were also elevated in fed LepR mutant fish. Expression levels of several hypothalamic genes involved in feed regulation were analyzed (*agrp*, *npy*, *orexin*, *cart-1*, *cart-2*, *pomc-a1*, *pomc-b*). No differences were detected between fed WT and mutants except for *pomc-b* (proopiomelanocortin-b), where levels were 7.5-fold higher in LepR fed mutants, suggesting that *pomc-b* expression is regulated by leptin signaling. Fatty acid (FA) content did not statistically differ in muscle of fed mutant fish compared to WT. However, fasted mutants exhibited significantly lower muscle FA concentrations, suggesting that LepR mutants exhibit increased FA mobilization during fasting. These data demonstrate a key role for leptin signaling in lipid and energy mobilization in a teleost fish.

## 1. Introduction

Leptin is a cytokine that has been linked to numerous physiological processes in vertebrates, though the hormone is largely known for regulating appetite and metabolism [1,2]. In mammals, leptin increases postprandially, suppresses appetite, and acts as an adipostat as it preferentially mobilizes lipids [3]. While leptin has been described as anorexigenic in many fishes, the hormone is primarily produced in the liver instead of fat, and there are reports of leptin regulating glucose homeostasis [4,5,6,7,8]. There are reported effects on lipid metabolism in some fishes where leptin stimulates lipolysis and increases lipid mobilization (grass carp, *Ctenopharyngodon idellus*; [9]; goldfish, *Carassius auratus*; [10]), plasma leptin levels increase with adiposity (rainbow trout, *Oncorhynchus mykiss*; [11]), and adipocytes secrete leptin (*O. mykiss*; [12]). Due to genome duplication, most fishes have two forms of leptin; leptin-a (LepA) and leptin-b (LepB), although some fishes like salmonids and cyprinids can have up to four paralogs (LepA1/LepA2, LepB1/LepB2). It appears the LepA and LepB paralogs have divergent functions that may be species specific [13]. Most studies are on LepA as it is the dominant form of leptin in most fishes and is typically more highly expressed than LepB [2]. There is low sequence conservation between the leptins of fishes with that of mammals; however, tertiary structure and ligand binding domains appear to remain highly conserved across vertebrates [14,15].

Leptin exerts its actions through the transmembrane leptin receptor (LepR), which is part of the class-1 helical cytokine receptor family [16,17]. After homodimerizing, the intracellular signaling is regulated through the highly conserved JAK/STAT pathway [18,19,20]. There is a single leptin receptor (LepR/LepRA1) characterized for nearly all vertebrates; however, recently a duplicated leptin receptor (LepRA2) has been documented for some fish including: rainbow trout (*O. mykiss*; [21]), Atlantic salmon (*Salmo salar*; [22]), Asian arowana (LepRa/b, *Scleropages formosus*; reviewed in [15]), European eel (*Anguilla Anguilla*), and Japanese eel (LepRa/b, *Anguilla japonica*; [23]). The second LepR present in salmon and trout is likely due to a salmonid specific genome duplication event that occurred around 25–100 million years ago, while the additional receptor present in the eel and arowana was suggested to have been derived from a separate and more ancient teleost 3R duplication event (~350 million years ago), and thereafter lost in the teleost line [15,21,22,23,24].

The different LepR paralogs and alternative receptor splice variants documented in fishes are regulated by the physiological state of the animal [21,25]. Teleost LepR’s are ubiquitously expressed, with high levels reported in the liver, gonad, and hypothalamus [2,21]. Leptin actions in the hypothalamus are not surprising due to this region of the brain being linked closely to regulating food intake [26]. There are various neuropeptides secreted from the hypothalamus including the orexigenic peptides; neuropeptide-Y (NPY), agouti-related peptide (AgRP), and orexin (OX, also called hypocretin). In addition, there are anorexigenic peptides, such as proopiomelanocortin (POMC) and cocaine- and amphetamine-regulated transcript (CART) that suppress food intake [27]. There are many reports of leptin actions on the hypothalamus to suppress feed consumption in mammals [28]. This typically occurs by leptin suppressing levels of *npy* and *agrp*, while increasing *pomc* and *cart* transcripts [28]. The studies in fishes examining leptin treatment on the hypothalamic feeding neuropeptides are more limited; however, generally the responses appear to be similar to that of mammals (reviewed in [8]). The paradigm is even more complex in fishes, especially due to the duplicated genes of many of the neuropeptides, particularly for the case of *cart*, where in medaka (*Oryzias latipes*; [29]) and sole (*Solea senegalensis*; [30]), there are six and seven genes documented respectively.

Leptin is encoded by the *obese* gene (*ob*) and the leptin receptor by the *diabetes* gene (*db*) in mammals [31,32]. Leptin deficient mice (*ob*/*ob*) that contain mutations in the leptin gene exhibit excessive hyperphagia and display a morbidly obese and diabetic phenotype [31,33]. Mice with mutations in the leptin receptor gene (*db*/*db*) show an almost indistinguishable phenotype to that of *ob*/*ob* mice, except that the *db*/*db* mice exhibit an increased degree of diabetes and some variation of fat distributions [33,34,35,36]. To date, the few studies in fishes investigating leptin or LepR deficiency have been conducted using zebrafish (*Danio rerio*) or medaka (*O. latipes*) as models, and they have displayed some variable phenotypes. LepR zebrafish mutants did not show increased adiposity, feeding, or hyperphagia, and instead displayed marked increases in insulin mRNA and other glucoregulatory factors [7]. By contrast, LepR deficient medaka did exhibit hyperphagia and food intake, which led to higher growth rates for post-juveniles and greater depots of visceral fat [37]. There have been varying reports across the teleost taxon regarding leptin physiology; it is possible that effects of leptin signaling may vary between species due to differences in life histories [8]. Further studies are required to elucidate the function of leptin in fishes as a group and individually by species.

In order to gain further insight into leptin physiology in teleost fish, we used CRISPR/Cas9 genome editing technology to disrupt expression of leptin receptors in rainbow trout (*O. mykiss*). By targeting the LepR genes, *lepra1* and *lepra2*, we aim to characterize how leptin signaling regulates feed intake, growth, and energy utilization in an important aquaculture species. Indices of growth performance, nutrient deposition, and hypothalamic gene expression were analyzed to characterize the phenotype of LepR mutants during satiable feed intake and fasting.

## 2. Materials and Methods

### 2.1. Animals

All procedures and research were approved and performed in accordance with the relevant guidelines and regulations by the Institutional Animal Care and Use Committee at the National Center for Cool and Cold Water Aquaculture (NCCCWA, protocol #177). Rainbow trout were maintained in buckets or tanks supplied with a flow-through water (temperature 12.5–13.5 °C, ambient photoperiod) and provided a commercially available feed (Zeigler Finfish G, Gardners, PA, USA; 42% protein, 16% fat).

### 2.2. CRISPR Gene Modification

The Alt-R CRISPR-Cas-9 System (Integrated DNA Technologies, IDT, Coralville, IA, USA) was used to genetically modify the leptin receptor genes, *lepra1* and *lepra2*, in rainbow trout following the previously established procedures [38,39] and utilizing the most recent genome assembly for this species [40]. To determine the exon/intron boundaries, the cDNA sequence was aligned with the corresponding genome sequence (*O. mykiss*, accession #GCA_013265735.3) using Splign (National Center for Biotechnology Information, NCBI, Bethesda, MD, USA; [41,42]). The CRISPR RNA (crRNA) sequence was designed using the IDT Custom Alt-R CRISPR-Cas9 guide RNA online program (Coralville, IA, USA) to target both leptin receptor genes (accession #’s, *lepra1*: JX878485 and *lepra2*: XM_021599667). The crRNA binding sites were immediately upstream of the receptor ligand binding domain to ensure disruption of leptin binding functionality. Using BLAST [41], the crRNA sequence, ATATCTCCCTGAGAGAGTGGCGG (sense), was compared to the rainbow trout genome to check for specificity for *lepra1* and *lepra2* genes and potential off-target effects. The crRNA (Alt-R crRNA XT, IDT) and tracrRNA (Alt-R, IDT) were diluted to 100 μM in the supplied IDTE buffer. The guide RNA (gRNA) solution was comprised of 3 μL of crRNA, 3 μL tracrRNA, and 94 μL Duplex buffer. The solution was heated at 95 °C for 5 min. The HiFi Cas9 nuclease (IDT) was diluted to 0.5 μg/μL in Cas9 working buffer (20 mM HEPES, 150 mM KCl, pH 7.5) and combined with an equal volume of gRNA solution. The ribonucleoprotein (RNP) complex was assembled by incubating the gRNA/Cas9 mixture at 37 °C for 10 min. Phenol red was added just prior to injection to visualize RNP delivery.

### 2.3. Microinjection and Early Rearing

Eggs and milt were collected on site from rainbow trout broodstock. Eggs were rinsed with milt activator solution (102.8 mM NaCl, 10 mM Tris, 20 mM glycine, pH 9.0, and 1 mM reduced glutathione added fresh), then fertilized and rinsed again. Embryos were held in activator solution at 10 °C and microinjected between 2.5–7 hr post-fertilization (before first cell division). During microinjection, embryos were placed in a tray and submerged in saline (0.9% NaCl), and 100–200 nL of the RNP complex was microinjected into the blastodisc. An equivalent number of embryos from each fertilization event were injected only with the Cas9 protein mixture; these fish were defined as wild type (WT). Immediately after injection, embryos were transferred to spring water and maintained in a 10–12 °C incubator. LepR mutants and WT fish were reared separately from hatching to approximately five months post-hatch (17.11 ± 0.26 g). At this point, all fish were anesthetized with tricaine methanesulfonate (100 mg/L; MS-222, Pentair Aquatic Eco-Systems, Apopka, FL, USA) and tagged with passive integrated transponders (PIT, Biomark Inc, Boise, ID, USA) that provided a unique ID for each fish. The adipose fins were clipped and stored in 70% ethanol for genotype analyses. To avoid tank effects on growth, all mutant and WT fish within the injection group were comingled and were provided feed at 1.5–2% biomass using automated feeders (Arvo-tec, Huutokoski, Finland). A subset of the comingled population was used in the subsequent phenotyping study described in Section 2.5.

### 2.4. Genotyping and Mutant Characterization

**Figure 1:**
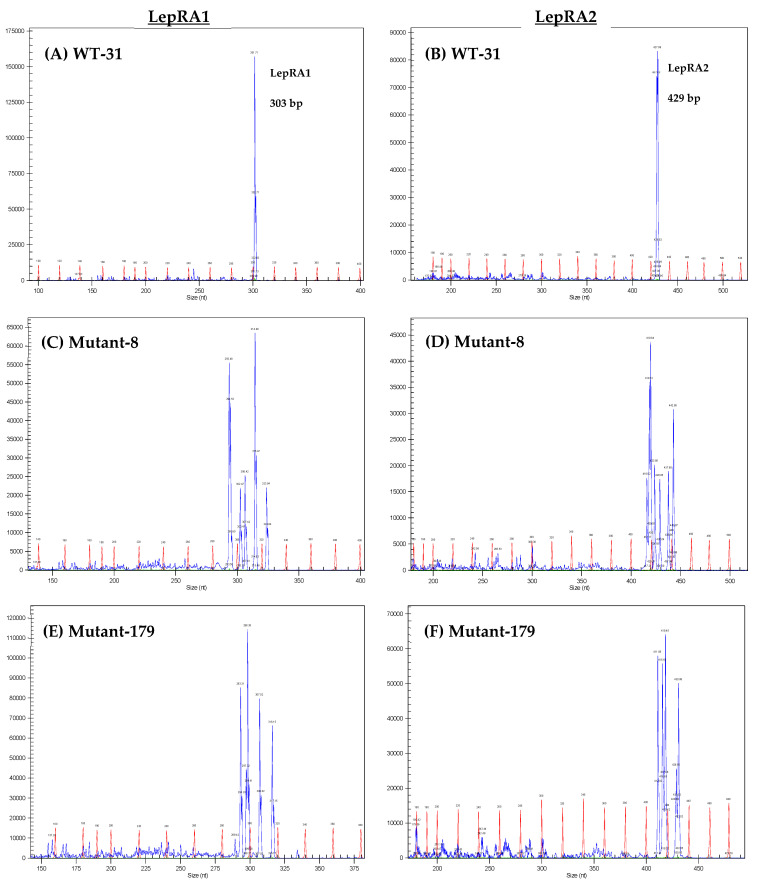
Representative wild type (WT) and mutant chromatograms that were generated using *lepra1* and *lepra2* gene specific primers for PCR amplification. Products were separated by capillary electrophoresis. Red peaks are the base pair (bp) ladder, and blue peaks represent the separated amplicons. Chromatograms show: (**A**) WT with intact *lepra1* peak ~303 bp, and (**B**) WT with intact *lepra2* peak at ~429 bp. Panels (**C**–**F**) are from example mutant fish, showing multiple peaks due to insertions/deletions, instead of a single peak for an intact gene.

Genomic DNA (gDNA) was extracted from individual fin clips using a Quick-DNA Miniprep kit (Zymo Research, Irvine, CA, USA). Tissue was mechanically homogenized in 0.5 mL of Genomic Lysis Buffer and then followed manufacturer’s protocol for column extractions. Samples were diluted to 25 ng/µL and polymerase chain reaction (PCR) amplified with the TaKaRa Taq protocol (TaKaRa Bio, Inc., Kusatsu, Shiga, Japan) using *lepra1* and *lepra2* specific primers (Table 1) that amplified gene regions surrounding the gRNA annealing site. The cycling parameters were 98 °C for 10 s, followed by 30 cycles of 98 °C for 10 s, 55 °C for 30 s, and then 72 °C for 60 s. The reverse primer of each set contained a WellRED fluorescent tag for amplicon detection on a GeXP Genetic Analysis System (Beckman Coulter, Brea, CA, USA) that separates the amplicons using capillary gene electrophoresis. Full-length intact amplicons were identified as single peaks at ~303 base pairs (bp) for *lepra1* and ~429 bp for *lepra2*. Individuals with successful gene mutagenesis (mutants) were identified with multiple peaks instead of a single peak, indicating mosaicism and the presence of insertions and deletions (indels) in the target genes (Figure 1). Only mutants without detectable intact amplicons were selected for the phenotyping study. The indels present in the *lepra1* and *lepra2* genes of each heterozygous mutant used in the phenotyping study were confirmed by sequencing the PCR amplicons with the Amplicon-EZ service from GENEWIZ (South Plainfield, NJ, USA; data not shown) and comparing them to the reference genome sequences. All of the mutant fish used for the phenotyping characterization study were part of the F0-parent generation and are therefore considered heterozygous mutants.

### 2.5. Phenotyping Study

After genotyping and identifying mutants with no detectable intact LepR genes, we performed a phenotyping study with a subset of these fish to characterize the LepR mutant phenotype. Rainbow trout (145.9 ± 3.08 g mean BW) were stocked 8–10 fish per tank into twelve 150-L tanks, which included six tanks of WT and six tanks of LepR mutants. Fish were allowed to acclimate for one week, during which feed was provided at 2% of tank biomass using automated feeders (Arvo-tec, Huutokoski, Finland). For the six-week phenotyping study, triplicate tanks for both WT and mutants were either fasted or fed to satiation. Satiation feed occurred by using automated feeders to provide a limited ration (1.25% of tank biomass), followed by daily hand feeding to satiation. This method ensured that all fish were indeed fed to satiation as feeding behavior could be monitored and it allowed availability of feed to all fish without overfeeding. Feed intake, body weights and lengths of all fish were recorded at the beginning of the study and at three and six weeks. Fed tanks were not provided feed on the day of sampling. Fish from all 12 tanks were sampled at three- and six-week timepoints (*n* = 4–5 fish/tank; *n* = 13–15 per treatment/timepoint). For sampling, fish were euthanized with a lethal dose of MS-222 (300 mg/L), length (cm) and weight (g) were recorded, and blood was collected from caudal vasculature using heparinized syringes. Blood glucose measurements were immediately analyzed using a Prodigy AutoCode glucometer (Prodigy Diabetes Care, LLC, Charlotte, NC, USA). Blood samples were centrifuged at 3000× *g* (4 °C) for 7 min to collect plasma that was aliquoted and stored at 80 °C. Whole liver tissue was removed and weighed to obtain the hepatosomatic index (HSI; (liver weight/total body weight) × 100). Similarly, the viscera were removed and weighed to calculate the viserosomatic index (VSI; (viscera weight/total body weight) × 100). Feed intake (FI) was calculated as the feed consumed/tank biomass × 100; feed conversion ratio (FCR) = feed consumed/g body weight gained; condition factor (CF) = (100,000× *g* body weight/mm length^3^); and specific growth rate (SGR) = (ln(final weight) − ln(initial weight))/days × 100. Liver, white muscle, visceral adipose tissues (~100 mg), and whole hypothalamus were collected and placed in 1 mL of RNAlater (Ambion Inc., Austin, TX, USA), kept overnight at 4 °C, and then stored at 80 °C until extractions. Additional pieces of liver, muscle, and fat were flash frozen in liquid nitrogen and then stored at 80 °C for fatty acid analysis.

### 2.6. Glycogen Analysis

Liver and muscle glycogen content were measured with a colorimetric glycogen assay kit following the manufacturer’s protocol (#MAK016, Sigma-Aldrich, St. Louis, MO, USA). Tissues were weighed (~60 mg/tissue) before homogenizing in 1 mL of ultrapure water and then boiled for 5 min. Samples were then centrifuged at 13,000× *g* for 5 min. For liver samples, the supernatant was diluted 1:25 with ultrapure water and 4 µL (fed fish) or 15 µL (fasted fish) were used in the assay in the assay. For muscle samples, supernatant volumes of 10 µL (fed fish) or 25 µL (fasted fish) were used in the assay. Variable volumes were used to maintain optical densities (OD) within the linear range of the standard curve. All samples and standards were run in duplicate. OD values were measured at 570 nm using a microplate reader (Varioskan, Thermo Scientific, Waltham, MA, USA). Glucose background and blank well ODs were subtracted from all wells to correct for background absorbance. Pooled plasma samples were run on each plate for interassay normalization. Adjusted OD values were used to interpolate concentrations from a five-point linear standard curve generated from standards run on each plate. Samples were normalized to the amount of tissue used in the assay.

### 2.7. Plasma Leptin and Cortisol ELISA

Plasma leptin was measured with a commercially available salmon leptin ELISA kit (Catalog #MBS935480; MyBioSource, Inc., San Diego, CA, USA). The assay was performed according to the manufacturers protocol. Briefly, plasma was diluted 1:75 in ultrapure water, followed by a 1:10 dilution with sample diluent. OD values were measured at 450 nm using a microplate reader (Varioskan, Thermo Scientific). Additional readings at 570 nm and blank well ODs were subtracted from all wells to correct for background absorbance. All samples and standards were run in duplicate. Pooled plasma samples were run on each plate for interassay normalization. Adjusted OD values were analyzed using non-linear regression and GraphPad Prism 8 (GraphPad, La Jolla, CA, USA), and were interpolated from a sigmoidal curve generated from standards on each plate.

Plasma cortisol was measured using a colorimetric Cortisol ELISA kit (#500360, Cayman Chemical, Ann Arbor, MI, USA). The assay was performed according to the manufacturers protocol. Steroids were extracted from 100 µL of plasma, and then reconstituted in 500 µL of ELISA buffer, of which 4 µL was used per well in the assay. OD values were measured at 420 nm using a microplate reader (Varioskan, Thermo Scientific). All samples and standards were run in duplicate. Pooled plasma samples were run on each plate for interassay normalization. A data analysis spreadsheet available from Cayman Chemical was used to analyze the data (www.caymanchem.com/analysis/elisa (accessed on 27 July 2021)). Adjusted OD values were used to interpolate concentrations from an eight-point linear standard curve generated from standards run on each plate.

### 2.8. Fatty Acid Analyses

Muscle, liver, and adipose samples from the six-week timepoint (*n* = 13–15 per treatment) were pulverized at liquid nitrogen temperature, and the powder was stored at −80 °C until fatty acid content was determined by fatty acid methyl ester (FAME) analysis. FAME derivatization was based upon a previously developed one-step method using acetyl chloride [43], as described in [44]. FAME were quantified by gas chromatography with flame ionization detection (GC-FID), as described previously [45].

### 2.9. RNA Isolation and Quantitative Real-Time PCR

Total RNA was extracted from tissues with 1 mL of Tri-Reagent (Molecular Research Center, Cincinnati, OH, USA) and RNA purification Direct-zol miniprep spin columns with DNase treatment using standard methods from the manufacturer (Zymo Research, Irvine, CA, USA). RNA quality was assessed by the presence of 18S and 28S ribosomal RNA bands using gel electrophoresis, and then quantified by absorbance OD 260:280 ratio using a Nanodrop 2000c spectrophotometer (Thermo Scientific, Waltham, MA, USA). Total RNA (1 µg) was used in a cDNA synthesis reaction via reverse transcription following the manufacturer’s instructions (Promega M-MLV Reverse Transcriptase, Madison, WI, USA). Messenger RNA (mRNA) levels of *lepa1*, *agrp*, *npy*, *orexin*, *cart1*, *cart2*, *pomca1*, *pomcb*, and *ef-1a* (elongation factor 1 alpha) were determined by qPCR using gene-specific primers (Table 1). When available, qPCR primer sequences were obtained from existing literature for rainbow trout or primer pairs were designed with Primer-3 and BLAST on NCBI [46]. Primers with other gene paralogs were compared to each other using BLAST to ensure no complementation.

All reactions were run in triplicate and performed on a QuantStudio 5 Real-Time PCR System (Applied Biosystems, Foster City, CA, USA), with the Applied Biosystems SYBR Green qPCR master mix, using 1.5 µM primers, and 2 µL of 1:6 diluted cDNA in a total reaction volume of 10 µL. The cycling parameters were 95 °C for 10 min, followed by 40 cycles of 95 °C for 30 s and 60 °C for 1 min. A dissociation melt curve step at the end was performed to verify a single PCR product. Negative controls included using water instead of an RNA template (no template control; NTC) and DNase treated RNA with no reverse transcriptase enzyme to ensure no genomic DNA contamination (no-amplification control; NAC). Cycle threshold (Ct) values for samples were transformed using a standard curve of serially diluted pooled cDNA versus Ct values (R^2^ = 0.97–0.99). Samples were then normalized to reflect the amount of template cDNA per ng total RNA loaded into each reaction (cDNA/ng total RNA). Data were also normalized to the expression levels of *ef-1a* as a secondary control to validate the total RNA normalization method (data not presented). The values are expressed as fold change relative to the mean of the three-week WT group, as indicated in the figure legends.

### 2.10. Statistical Analyses

The fatty acid data (six-week only) were analyzed by one-way analysis of variance (ANOVA, WT × mutant) for fed and fasted groups separately using PC-SAS (Version 9.2, Cary, NC, USA). All other data were analyzed by two-way ANOVA (genotype × time) and were analyzed for significance at each time point with Fisher’s least significant difference (LSD) test. These analyses were performed using GraphPad Prism 8 (GraphPad, La Jolla, CA, USA). The level set for statistical significance for all analyses was *p* < 0.05 and data are shown as mean values ± SEM.

## 3. Results

### 3.1. Growth and Feeding Response to LepR Deficiency

**Figure 2:**
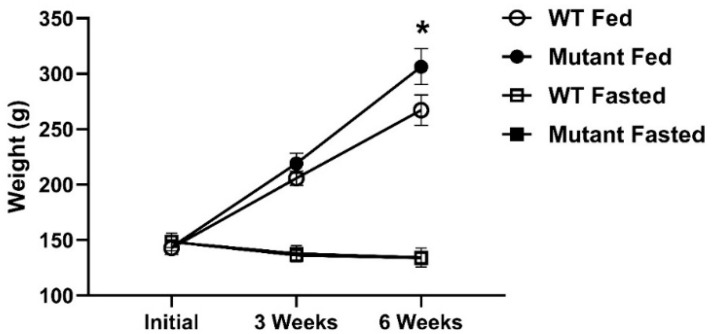
Body weight (g) over time from WT and LepR mutant rainbow trout that were fed to satiation or fasted for six weeks. Black and white squares for fasted groups are superimposed as there were no differences in weight. * denotes significant difference between WT and mutant fed fish at six weeks (*p* = 0.03, *n* = 12–15; initial and three week sampling *n* = 26–30).

Using CRISPR/Cas9 gene editing, we successfully generated rainbow trout with mutations in the *lepra1* and *lepra2* genes. Mutations were confirmed via PCR and capillary gel electrophoresis. The resultant chromatograms showed various indels as multiple amplicons (peaks) for both genes in an individual, while WT fish had only a single peak representing the intact gene (Figure 1). We exposed LepR mutant (lacking any detectable intact gene peak) and WT fish to a six-week period of either fasting or satiation feeding. All data presented herein are from the phenotyping study. There were no differences in weight between fasted WT and mutant fish (Figure 2). Other sampling data (length, viscerosomatic index; VSI, and hepatosomatic index; HSI) were not significantly different between fasted fish at either timepoint (Supplemental Figure S1). With the exception of FA data, we have provided results from fasted fish in the supplemental data and will primarily present results and discussion on the differential phenotype of the satiation fed LepR mutant and WT fish.

**Figure 3:**
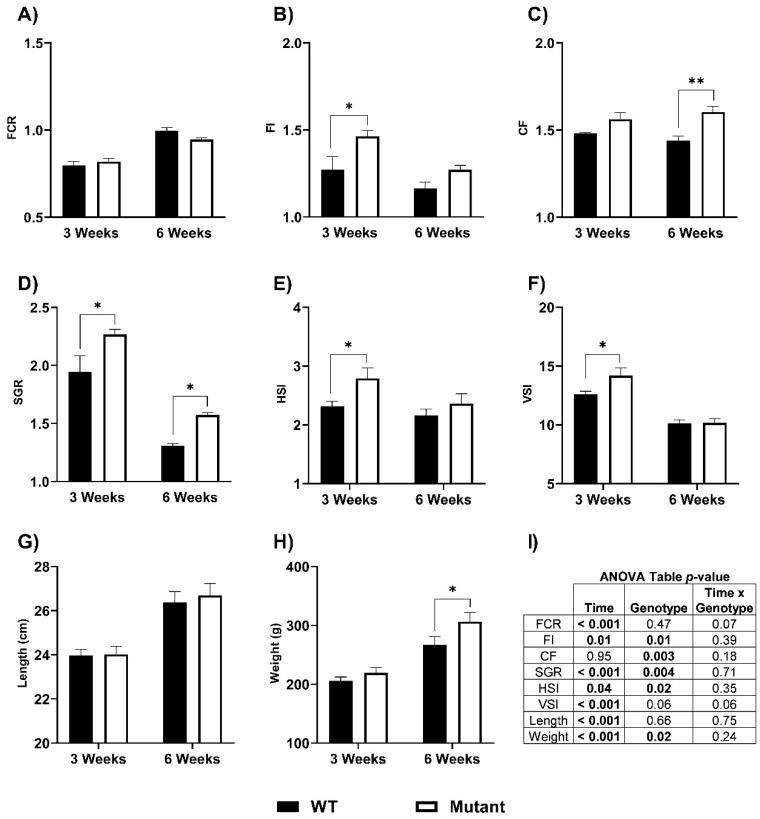
Growth and feeding responses of WT and LepR mutant rainbow trout that were fed to satiation for six weeks. (**A**) feed conversion ratio (FCR), (**B**) feed intake (FI), (**C**) condition factor (CF), (**D**) specific growth rate (SGR), (**E**) hepatosomatic index (HSI), (**F**) viscerosomatic index (VSI), (**G**) length (cm), (**H**) weight (g), and (**I**) ANOVA table. Values reported as means ± SEM. * denotes significant differences between WT and mutant fed fish within each time point. (* *p* ≤ 0.04, ** *p* = 0.004; HSI, VSI, length, weight, *n* = 13–28; FCR, FI, CF, SGR, *n* = 3 tanks per genotype). The significant *p*-values from different interactions are shown bolded in the ANOVA table in panel **3I.**

Growth, feed intake, and body morphometric responses for the fed LepR mutant and WT fish from the phenotyping study are shown in Figure 3. There was a significant main effect of genotype (WT vs. mutant) on feed intake (FI), condition factor (CF), specific growth rate (SGR), and HSI (Figure 3I), although pairwise comparisons between WT and LepR mutants within a time period were not always significantly different. LepR mutant fish exhibited greater FI (three weeks; *p* = 0.02; WT: 1.27 ± 0.07%, mutant: 1.47 ± 0.03%, and six weeks; *p* = 0.14; WT: 1.16 ± 0.04%, mutant: 1.27 ± 0.03%). The hyperphagia led to heavier body weight in mutants (three weeks; *p* = 0.30; WT: 205.69 ± 6.51 g, mutant: 219.16 ± 9.11 g, and six weeks; *p* = 0.03; WT: 267.17 ± 13.76 g, mutant: 306.41 ± 16.10 g), faster SGR (three weeks; *p* = 0.02; WT: 1.94 ± 0.14%, mutant: 2.27 ± 0.05%, and six weeks; *p* = 0.04; WT: 1.31 ± 0.02%, mutant: 1.57 ± 0.02%), and greater CF (three weeks; *p* = 0.08; WT: 1.48 ± 0.01%, mutant: 1.57 ± 0.04%, and six weeks; *p* = 0.004; WT: 1.44 ± 0.03%, mutant: 1.60 ± 0.03%). Mutant fish also displayed increases in VSI at three weeks (*p* = 0.01; WT: 12.60 ± 0.26%, mutant: 14.18 ± 0.66%, and six weeks; *p* = 0.97; WT: 10.14 ± 0.27%, mutant: 10.16 ± 0.36%) and HSI at three weeks (there weeks; *p* = 0.03; WT: 2.32 ± 0.08%, mutant: 2.79 ± 0.18%, and six weeks; *p* = 0.31; WT: 2.16 ± 0.11%, mutant: 2.36 ± 0.17%). Fork length (cm) and feed conversion ratio (FCR) were similar between WT and LepR mutants (Figure 3).

### 3.2. Glycogen, Cortisol, and Leptin Responses

**Figure 4:**
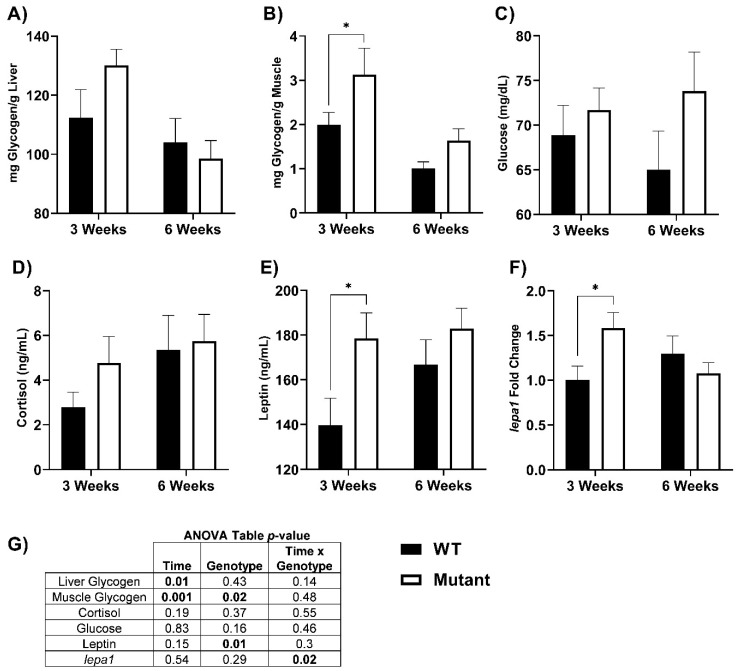
Liver and muscle glycogen, cortisol, and leptin measurements in WT and LepR mutant rainbow trout that were fed to satiation for six weeks. (**A**) liver glycogen (mg/g liver), (**B**) muscle glycogen (mg/g muscle), (**C**) blood glucose (mg/dL), (**D**) plasma cortisol (ng/mL), (**E**) plasma leptin (ng/mL), (**F**) hepatic *lepa1* mRNA expression (fold-change from three-week WT), (**G**) ANOVA table. Values reported as means ± SEM. * denotes significant differences between WT and mutant fed fish within each time point. (* *p* ≤ 0.03, *n* = 8–15). The significant *p*-values from different interactions are shown bolded in the ANOVA table in panel **4G.**

Glycogen content of muscle and liver, blood glucose, plasma cortisol, plasma leptin, and *lepa1* gene expression in fed LepR mutant and WT fish is shown in Figure 4. Muscle glycogen content was higher in LepR mutant fish (three weeks; *p* = 0.03; WT: 1.99 ± 0.28, mutant: 3.12 ± 0.60 mg/g muscle, and six weeks; *p* = 0.20; WT: 1.01 ± 0.15, mutant: 1.64 ± 0.26 mg/g muscle). However, liver glycogen did not differ between mutant and WT fish (three weeks; *p* = 0.13; WT: 112.38 ± 9.55, mutant: 130.16 ± 5.38 mg/g liver, and six weeks; *p* = 0.60; WT: 103.99 ± 8.18, mutant: 98.56 ± 6.07 mg/g liver). Blood glucose (three weeks; *p* = 0.65; WT: 68.88 ± 3.34, mutant: 71.69 ± 2.46 mg/dL, and six weeks; *p* = 0.10; WT: 65.00 ± 4.34, mutant: 73.80 ± 4.38 mg/dL) and plasma cortisol (three weeks; *p* = 0.33; WT: 2.79 ± 0.67, mutant: 4.77 ± 1.18 ng/mL, and six weeks; *p* = 0.81; WT: 5.34 ± 1.55, mutant: 5.74 ± 1.19 ng/mL) were not significantly different at either timepoint, nor was a main effect of genotype detected. Hepatic *lepa1* transcript levels were higher in mutant fish at three weeks (*lepa1*; *p* = 0.02; WT: 1.00 ± 0.16, mutant: 1.58 ± 0.18 ng/mL, and six weeks; *p* = 0.34; WT: 1.29 ± 0.20, mutant: 1.08 ± 0.12 ng/mL). Plasma leptin was elevated in mutant fish, although pairwise comparisons detected a significant difference only at three weeks (three weeks; *p* = 0.02; WT: 139.62 ± 12.15, mutant: 178.58 ± 11.35 ng/mL, and six weeks; *p* = 0.29; WT: 166.82 ± 11.13, mutant: 182.93 ± 9.05 ng/mL).

### 3.3. Hypothalamic mRNA Response

**Figure 5:**
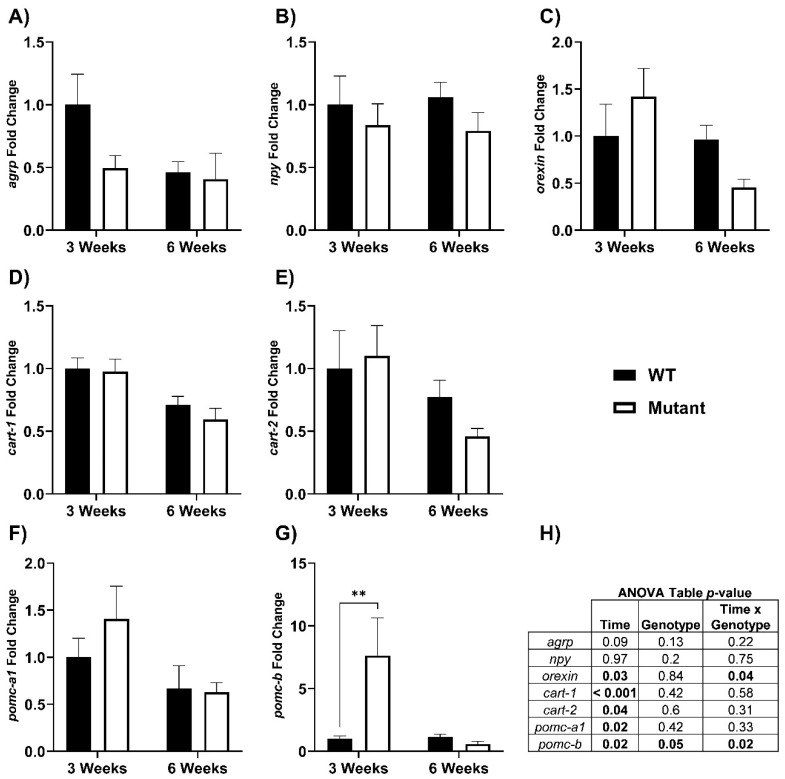
Hypothalamic genes involved in feed intake were measured in WT and LepR mutant rainbow trout that were fed to satiation for six weeks. (**A**) *agrp*, (**B**) *npy*, (**C**) *orexin*, (**D**) *cart-1*, (**E**) *cart-2*, (**F**) *pomc-a1*, (**G**) *pomc-b*, (**H**) ANOVA table. Values reported as means ± SEM and expressed as fold change from the three-week WT. ** denotes significant differences between WT and mutant fed fish within each time point. (** *p* = 0.004, *n* = 9–15). The significant *p*-values from different interactions are shown bolded in the ANOVA table in panel **5H.**

A range of hypothalamic genes involved in regulating feed intake were measured in WT and LepR mutant rainbow trout. The only transcript that exhibited a significant fold change difference between WT and mutants was *pomc-b*, where levels were ~7.5-fold higher in mutant fish at three weeks (Figure 5: three weeks; *p* = 0.004; WT: 1.00 ± 0.24, mutant: 7.64 ± 2.99, and six weeks; *p* = 0.79; WT: 1.13 ± 0.22, mutant: 0.59 ± 0.19). None of the other genes measured (*agrp*, *npy*, *orexin*, *cart-1*, *cart-2*, *pomc-a1*) in the hypothalamus were different between fed WT and LepR mutant fish. Similarly, fasted fish exhibited no marked differences in any of the hypothalamic genes except for at three weeks *npy* mRNA was higher (*p* = 0.007; WT: 0.93 ± 0.19, mutant: 1.54 ± 0.17) and *cart-1* was lower in mutant fish (Supplemental Figure S3: *p* = 0.02; WT: 1.12 ± 0.11, mutant: 0.76 ± 0.10).

### 3.4. Fatty Acid Profiles

Individual fatty acid (FA) profiles and major classes were quantified in muscle, liver, and adipose tissues for WT and LepR mutant fed and fasted fish. There were no consistent differences in FA concentrations of liver and adipose tissues (Supplemental Tables S1 and S2). There were no differences in muscle FAs between fed LepR mutant fish compared to fed WT, in part due to the high variability in the results (Table 2). In contrast, all FAs were significantly lower in fasted mutant fish compared to WT fasted fish (*p* = 0.002–0.035), with the exception of the odd-chain fatty acid 17:0 (*p* = 0.173; WT: 88.5 ± 6.1, and mutant; 76.2 ± 6.3 µg/g) and 21:0 (*p* = 0.830; WT: 41.0 ± 4.6, and mutant: 39.7 ± 4.2 µg/g). Collectively, the muscle of fasted mutants exhibited approximately 27% lower adiposity (total FA) compared to WT muscle.

## 4. Discussion

Leptin is extensively researched in mammals and the hormone is classically described as an adipostat; however, the understanding of leptin physiology and hormone interactions in non-mammalian vertebrates remains unclear [8]. By generating mutant rainbow trout deficient in leptin signaling, we examined the impact of leptin and its receptors upon the regulation of feeding, growth, and energy utilization in a teleost fish. We compared the phenotypes of LepR mutant and WT rainbow trout that were either fed to satiation or fasted for six weeks. The LepR mutants exhibited a hyperphagic phenotype, which led to heavier body weight, faster specific growth rate, and increased muscle glycogen stores, as well as increased muscle fatty acid mobilization during fasting. In mammals, leptin (*ob*/*ob*) and leptin receptor (*db*/*db*) deficiencies lead to excessive hyperphagia, morbid obesity, and a diabetic phenotype [31,33]. While we recorded significant increases in feed intake and growth, the degree of hyperphagia and weight gain observed for the LepR mutant trout in this study was not as extreme as the phenotype documented for mammals. This could be due to the difference in feeding behaviors between these groups of vertebrates, as fishes can show high variability in food intake with large opportunistic meals followed by delays in subsequent feeding until gut processing [47,48]. It is possible that the increased feeding effects from impaired leptin signaling cannot compensate for these innate behaviors. Furthermore, caloric inputs, basal energy requirements, and metabolic regulation of non-mammalian vertebrates can differ substantially from that of homeotherms of equivalent body size, and thus could lead to some variations in responses between these vertebrate classes [49,50]. Interestingly, LepR mutant medaka exhibited hyperphagia, but this did not translate into increased growth for adult fish [37]. However, in the same study, the younger, post-juvenile (five weeks post-hatch) LepR deficient medaka showed greater body size and condition factor compared to WT fish [37]. The rainbow trout in the present study were considered juveniles and sexually immature. Perhaps, in fish, there is a relationship between stage of development and the severity of the hyperphagia and growth benefit in LepR mutants. Another study concluded that the effects of leptin signaling on food intake and digestion in LepR mutant zebrafish may primarily occur during early development; however, no significant differences in growth were recorded during larval or juvenile phases [51].

Body weight, SGR, CF, HSI, and VSI were all greater in fed LepR mutants, and were likely elevated in part due to the increased FI for these fish. There were a few factors—FI, HSI, and VSI—that were significantly higher at three weeks, but the differences between the groups were not significant at six weeks. The attenuated hyperphagia at six weeks, and subsequent effect on other response variables, are likely due in part to reduced feeding behavior in both genotypes caused by low fish density (4–5 fish per tank, ~6 kg m^−3^) after removing fish during the three-week sampling. Studies have shown that rearing rainbow trout at lower tank densities (10 kg m^−3^) can affect feeding behavior as a non-competitive feeding strategy can develop and result in increased size variability [52]. We did not observe any significant heterogeneity in SGR within any tanks, suggesting equitable feed intake among individuals within tanks. This was feasibly due to the small size of the fish (~150 g) and the short six-week timeframe of the study. Additionally, the reduction in feed intake at six weeks is likely related to the increased leptin levels in WT fish at this timepoint. Therefore, since the mutant phenotype is largely dependent on hyperphagia, the overall reduction in feed intake after the three-week sampling likely attenuated the phenotypic differences between the two treatment groups.

In the absence of functioning leptin receptors, we show increased plasma leptin and corresponding hepatic *lepa1* levels. The elevated measures of leptin that paired with leptin receptor deficiency did not translate into significantly elevated blood glucose for fed LepR mutants, despite higher blood glucose concentrations in these fish. Elevated whole-body glucose has previously been reported for larval zebrafish LepR mutants [7]. Previous studies have shown a relationship between leptin and glucose regulation in fishes; where treatment with exogenous leptin induces hyperglycemia in goldfish, (*C. auratus*, [53]), rainbow trout (*O. mykiss*, [5]), and tilapia (*Oreochromis mossambicus*, [6]; *Oreochromis niloticus*, [54]). The increased glycemia observed in many fishes is accompanied by a decline in hepatic glycogen, suggesting stimulation of glycogenolysis and a role in mobilizing carbohydrates [2]. We found increased levels of muscle glycogen in fed LepR mutant fish, but no differences in hepatic glycogen (higher but not significant) content at three-weeks. These data indicate that leptin receptor deficiency in rainbow trout leads to increased carbohydrate stores, primarily in the muscle with no hyperglycemia, corroborating leptin’s role in glucose mobilization.

HSI can be indicative of metabolic status and overall energy stores reflective of recent food consumption, as fishes can readily store energy in the form of glycogen in the liver [55]. HSI and VSI were both higher in fed LepR mutant fish. The higher HSI may correspond to both higher feed intake and increased liver glycogen levels at three-weeks for fed LepR mutants. In addition, we quantified VSI as a proxy for visceral fat depots. Similarly, the VSI of fed LepR mutant trout were higher than that of WT fish at three weeks, suggesting an increase in visceral adiposity, although this response did not extend to six weeks. The increased HSI and VSI are likely due to the additional food consumed and thus the increased energy storage by the LepR mutant trout; however, it could result from a direct effect of impaired leptin signaling on mechanisms regulating nutrient storage and partitioning. Interestingly, mutant LepR zebrafish do not show any increase in adiposity [7]; however, adult LepR mutant medaka exhibited increased VSI and larger visceral fat depots compared to WT fish [37].

In mammals and fishes, leptin signals within the brain reduce food consumption, typically by suppressing levels of the orexigenic hormones *npy* and *agrp*, while increasing anorexigens like *pomc* and *cart* [8,28]. We measured a range of these hypothalamic genes known to be involved in regulating food intake in rainbow trout (*agrp*, *npy*, *orexin*, *cart-1*, *cart-2*, *pomc-a1*, *pomc-b*) and we hypothesized they would be regulated in the absence of leptin signaling. No differences were detected between fed WT and mutants for any of the genes measured, except for *pomc-b*, where levels were 7.5-fold higher in LepR mutants at three weeks and returned to WT levels by six weeks (Figure 5G). These findings suggest that leptin signaling in the brain could be mediated in part through *pomc-b*. By contrast, leptin deficient (*ob*/*ob*) mice have significantly lower hypothalamic *pomc* mRNA compared to WT and leptin treated *ob*/*ob* mice [56]. Similarly, LepR mutant medaka have increased levels of *npy-a* and *agrp*, with decreased *pomc-1* in the diencephalon region of the brain [37]. Additionally, a brain transcriptomic analysis with LepR mutant zebrafish shows decreases in many anorexigenic genes, including *pomc*, and a leptin-dependent negative correlation between *pomc* and cannabinoid receptor 1 (*cnr1*) expression [57].

Many neuropeptides have duplicated genes with different patterns of expression in fishes, leading to a more complex paradigm compared to mammals [27]. There are three *pomc* genes (*pomc-a1*, *pomc-a2*, and *pomc-b*) and one splice variant (*pomc-a2s*) documented for rainbow trout [58]. The POMC molecule in fishes is a common precursor that is post-translationally cleaved to form melanocyte-stimulating hormone (α-, β-MSH; ɣ-MSH was lost in the teleost lineage), adrenocorticotropic hormone (ACTH), β-endorphin (β-END), and β-lipotropic (β-LPH) hormones [27,59]. We did not detect changes in *pomc-a1* (Figure 5F), and hypothalamic levels of *pomc-a2* were below detection limits (data not shown). Initial studies from rainbow trout and barfin flounder predicted that the *pomc-b* isoform had potentially lost its role in energy homeostasis [58,60]. However, a different study suggests *pomc-b* is the most important variant regarding feed intake in Atlantic salmon [61]. This agrees with the data from the present study, where *pomc-b* was the only neuropeptide and *pomc* variant that was regulated in the absence of leptin signaling with satiation feeding. Typically, *pomc* transcripts are stimulated by leptin to reduce food intake in vertebrates [8,28,56]. This has also been shown for rainbow trout, where leptin injection stimulated transcription of all three *pomc* genes [62]. Conversely, in mandarin fish (*Siniperca chuatsi*), LepA treatment decreased *pomc* expression two-hours post-injection, but did not coincide with a reduction in feed intake [63]. It is confounding that in the current study, none of the feeding neuropeptides were altered in the absence of leptin signaling except for *pomc-b*, an anorexigenic peptide which was substantially elevated. Possibly, a different factor was regulated by the increased feeding in LepR mutants or the hyperphagia itself, that in turn stimulated *pomc-b* synthesis. Desacetyl-α-MSH was shown to stimulate lipid mobilization in rainbow trout, and it was suggested that it could be a candidate by which leptin could regulate fat metabolism [64,65]. Perhaps there is a connection here with the upregulation of *pomc-b* in our LepR mutant rainbow trout and downstream α-MSH. Interestingly, recombinant LepA1 treatment stimulated brain levels of *pomc-a1* but had no effect on *pomc-b* in Atlantic salmon [66]. In fact, neither a long-term (20 days) nor acute (4 h) leptin treatment had a significant effect on *pomc-b* levels in salmonids [4,66]. In fishes, there is much still to realize about the role of leptin regulating food intake in the brain and understanding the variable responses observed across taxa. With the CRISPR/Cas-9 tools for gene editing becoming more accessible, particularly in those non-traditional model species, continued studies on the leptin/LepR loss of function will help shed light on some of the variables controlling food intake in fishes.

In mammals, leptin stimulates lipolysis and can regulate FA homeostasis in non-adipocyte cells [67,68]. There are also numerous reports of leptin regulating lipid metabolism by stimulating lipolysis and decreasing lipogenic factors in fishes (reviewed in [69]). In this study, we analyzed a wide range of FAs within muscle, liver, and adipose tissues. Almost no changes were detected in the FA levels of liver and adipose tissues (Supplemental Tables S1 and S2); however, concentrations of total FA in muscle of fed mutant fish was 15% higher compared to WT muscle, albeit not significantly higher (Table 2). These data suggest that, in addition to increased muscle carbohydrates, fed LepR mutant trout may have increased FA deposition in this tissue. These findings are consistent with previous reports in fish and mammals reporting the lipolytic effects of leptin. A previous study in rainbow trout adipocytes demonstrated that leptin stimulates lipolysis and decreases FA uptake [70]. There are reports in mammals where leptin directly regulates lipid metabolism in muscle by partitioning FAs toward oxidation and away from storage as triacylglycerol [71]. Additionally, *ob*/*ob* mice contain greater levels of intermuscular fat, likely due to the regulation of lipogenesis in the muscle by insulin [72]. Without a functional leptin receptor and under a satiation feeding regime, the rainbow trout in the current study exhibited increased energy storage (both FAs and glycogen) in white muscle. Interestingly, LepR mutant medaka have increased visceral fat depots; however, there were no increases in fat deposits in muscle or liver tissue [37]. At this stage, it is unclear whether the possible increased energy stores in muscle are due to indirect effects of increased feed intake or direct effects of impaired leptin signaling on nutrient partitioning mechanisms. It is also possible that there are other endogenous glucoregulatory factors (i.e., insulin) in vivo that are also compensating for the disruption in leptin signaling in rainbow trout. In mammals, leptin can inhibit insulin-stimulated lipogenesis [68]. We did not measure insulin in the present study, although this is an interesting area regarding leptin biology in fishes to investigate further. There are recent reports of leptin transcripts upregulated directly by glucose and insulin in tilapia, where there appears to be a conserved leptin–insulin axis [73,74].

Interestingly, fasted LepR mutants exhibited significantly lower muscle FA content compared to WT fish for virtually all FA species analyzed, including total FA, suggesting that fasted LepR mutants exhibit increased FA mobilization during fasting. Fasted LepR mutants also depleted liver glycogen stores faster than WT fish (Supplemental Figure S2A), which is likely also linked with lower plasma glucose in LepR mutants at three weeks. These findings provide strong support for the concept that leptin plays a significant role regulating energy mobilization during nutrient insufficiency. In mammals, there is evidence that during fasting, a reduction in plasma leptin mediates a shift in the reliance upon glycogenolysis to lipolysis to maintain availability of glucose precursors under catabolic conditions [75]. Possibly, a glucose–FA metabolic cycle in conjunction with insulin is key to understanding the fasted LepR mutant phenotype in rainbow trout. Both *ob*/*ob* mice and LepB mutant zebrafish exhibited dysregulation of arachidonic acid (20:4), suggesting that there is a connection across vertebrate groups regarding leptin and FA metabolism [76]. We have shown previously that there are two leptin receptor paralogs (LepRA1 and LepRA2) in rainbow trout, and that they are differentially expressed across tissues and during fasting [21]. The *lepra1* receptor was significantly upregulated in white muscle by seven-days of fasting, and transcripts continued to rise five-fold at 14-days [21], providing support for elevated leptin signaling in muscle during fasting. This result being in concordance with the present study suggests that during fasting, leptin regulates the mobilization of lipids from muscle, primarily through LepRA1. However, since we generated a rainbow trout knockout that was deficient in both *lepra1* and *lepra2*, we are unable to clearly identify receptor-specific functions.

In conclusion, these studies demonstrate that leptin signaling plays a key role in regulation of appetite and nutrient partitioning during feeding, and nutrient mobilization during fasting in a teleost fish. These studies are the first to characterize the phenotype of leptin receptor-deficient rainbow trout created using the CRISPR/Cas-9 method for gene editing. LepR mutants exhibited a hyperphagic phenotype, which led to heavier body weight, faster specific growth rate, increased HSI and VSI, and greater condition factor. This phenotype was associated with a marked increase in hypothalamic *pomc-b* mRNA. The LepR mutants also exhibited increased energy stores in muscle, an aspect of leptin biology conserved from mammalian models. Leptin clearly plays a key role in food intake, energy storage and mobilization in rainbow trout; however, additional studies on adipose- and glucoregulatory pathways in future generations of homozygous mutants are warranted to characterize the mechanisms responsible for the phenotype of the LepR deficiency.

## Figures and Tables

**Table 1:** List of primer sets used for genotyping polymerase chain reaction (PCR) and quantitative PCR (qPCR) in rainbow trout.

PCR Primers				
Gene	Forward (5′-3′)	Reverse (5′-3′)	Accession #	Product Size (bp)
*lepra1*	TGTGATGTGGTTATTAGCCTGT	ACATCTCCCCAAAAGGCTGG	JX878485	303
*lepra2*	CAACTGCCAGTCAGGTGATCTT	TGCATATTACTGTTGACCCATCC	XM_021599667	429
**qPCR Primers**				
Gene	Forward (5′-3′)	Reverse (5′-3′)	Accession #	Reference
*lepa1*	GGTGATTAGGATCAAAAAGCTGGA	GACGAGCAGTAGGTCCTGGTAGAA	AB354909	Salmerón et al., 2015
*agrp*	ACTCACCGACGATTCCTTCC	CAGCTGCATCACCTCAGCAA	XM_036964549	−
*npy*	TGAAGGAGAGCACAGACACG	TGAGATCAGTTGCTCGTCGC	AF203902	−
*orexin*	ATGACGCAGCCACTGGAATC	TGACCCGTGGAGTAGCTGAT	KR080508	Gong et al., 2016
*cart-1*	GCTCCCCTGAAGACCCCATA	CATCAGCATCACACATGGGAA	XM_021564309	−
*cart-2*	CGCTGCTGTCCATCCTATGT	TCGTGTAGAGCTCCAAGAAGAC	XM_021573503	−
*pomc-a1*	CTCGCTGTCAAGACCTCAACTCT	GAGTTGGGTTGGAGATGGACCTC	X69808	Leder and Silverstein, 2006
*pomc-b*	CCAGAACCCTCACTGTGACGG	CCTGCTGCCCTCCTCTACTGC	X69809	Leder and Silverstein, 2006
*ef-1* *α*	CATTGACAAGAGAACCATTGA	CCTTCAGCTTGTCCAGCAC	AF498320	Cleveland and Weber, 2014

*lepra1*/*lepra2*: leptin receptor A1/A2; *lepa1*: leptin A1; *agrp*: agouti-related peptide; *npy*: neuropeptide Y; *cart-1*/*cart-2*: cocaine- and amphetamine-regulated transcript-1/2; *pomc-a1*/*pomc-b*: proopiomelanocortin-a1/b; *ef-1α*: elongation factor 1 alpha; bp: base pairs; Accession #: GenBank accession number.

**Table 2:** Fatty acid content (µg/g tissue) of muscle tissue from WT and LepR mutant rainbow trout that were fed to satiation or fasted for six weeks. Data shown is from the final sampling at six weeks, and values are reported as means ± SEM. * and bold values denote a significant difference between WT and mutant fish groups (*n* = 15).

Muscle						
Fatty Acid	WT Fed	Mutant Fed	*p*-Value	WT Fasted	Mutant Fasted	*p*-Value
14:0	467.1 ± 46.5	548.2 ± 54.4	0.270	334.9 ± 31.6	221.2 ± 23.7 *	**0.010**
16:0	4703.4 ± 415.9	5309.5 ± 443.3	0.329	3855.8 ± 240.9	2811.7 ± 185.0 *	**0.002**
17:0	91.8 ± 5.7	97.9 ± 7.6	0.529	88.5 ± 6.1	76.2 ± 6.3	0.173
18:0	1287.9 ± 102.3	1493.9 ± 129.3	0.226	1240.6 ± 76.9	919.3 ± 58.7 *	**0.003**
20:0	51.0 ± 4.1	58.4 ± 4.8	0.255	45.1 ± 3.1	33.6 ± 3.0 *	**0.013**
21:0	40.4 ± 3.6	41.8 ± 4.4	0.807	41.0 ± 4.6	39.7 ± 4.2	0.830
22:0	36.3 ± 3.2	43.6 ± 4.1	0.175	28.8 ± 2.1	20.5 ± 1.8 *	**0.006**
SFA	6814.6 ± 577.7	7731.7 ± 645.8	0.301	5772.0 ± 358.2	4243.7 ± 278.7 *	**0.003**
16:1*n*-7, Z	1244.9 ± 148.5	1479.2 ± 150.2	0.278	867.7 ± 79.0	571.4 ± 64.5 *	**0.009**
18:1*n*-9, E	48.5 ± 3.8	55.5 ± 4.9	0.268	43.3 ± 2.8	30.3 ± 2.3 *	**0.002**
18:1*n*-9, Z	5953.5 ± 666.9	7134.2 ± 705.4	0.236	4971.6 ± 425.2	3336.8 ± 341.4 *	**0.007**
18:1*n*-7, Z	708.8 ± 71.9	809.3 ± 78.3	0.355	573.9 ± 46.4	394.6 ± 36.7 *	**0.006**
20:1*n*-9	406.3 ± 45.8	479.2 ± 45.4	0.269	381.6 ± 35.6	269.8 ± 30.0 *	**0.026**
22:1*n*-9	61.1 ± 6.5	71.1 ± 7.1	0.308	62.7 ± 5.4	43.3 ± 4.5 *	**0.012**
24:1*n*-9	60.7 ± 4.8	69.6 ± 5.9	0.251	62.9 ± 4.7	48.3 ± 3.2 *	**0.019**
MUFA	8534.7 ± 949.8	10,155.9 ± 999.0	0.251	7002.2 ± 595.7	4722.0 ± 481.4 *	**0.007**
18:2*n*-6, Z, Z	4693.1 ± 463.9	5237.4 ± 488.2	0.427	3843.6 ± 313.0	2696.0 ± 235.0 *	**0.008**
18:3*n*-6	94.7 ± 8.2	119.0 ± 9.7	0.068	67.7 ± 4.9	49.4 ± 4.8 *	**0.013**
18:3*n*-3	492.3 ± 45.3	549.3 ± 50.1	0.409	393.2 ± 28.1	277.4 ± 23.0 *	**0.004**
20:2*n*-6	363.5 ± 29.4	408.6 ± 36.9	0.352	293.1 ± 23.3	212.3 ± 17.6 *	**0.012**
20:3*n*-6	236.2 ± 11.9	257.1 ± 23.1	0.438	206.6 ± 11.3	162.5 ± 9.3 *	**0.007**
20:3*n*-3	41.9 ± 3.5	45.9 ± 4.1	0.475	34.4 ± 2.4	25.3 ± 1.8 *	**0.007**
20:4*n*-6	308.8 ± 15.4	349.3 ± 22.9	0.159	339.2 ± 13.0	298.2 ± 11.7 *	**0.029**
22:2*n*-6	32.4 ± 3.1	37.3 ± 3.7	0.326	28.5 ± 2.6	20.5 ± 2.1 *	**0.024**
20:5*n*-3	462.8 ± 22.0	491.1 ± 30.3	0.462	513.5 ± 17.1	457.8 ± 16.1 *	**0.027**
22:4*n*-6	42.0 ± 2.4	48.9 ± 3.7	0.134	36.6 ± 1.8	28.1 ± 1.8 *	**0.003**
22:5*n*-6	111.6 ± 5.4	128.6 ± 7.7	0.086	99.3 ± 4.5	85.2 ± 4.4 *	**0.035**
22:5*n*-3	142.6 ± 6.4	152.6 ± 10.8	0.445	161.1 ± 7.0	127.4 ± 7.0 *	**0.002**
22:6*n*-3	2778.0 ± 96.9	2904.4 ± 146.2	0.484	3368.6 ± 125.2	2965.2 ± 68.6 *	**0.012**
PUFA	9800.1 ± 693.5	10,729.4 ± 807.1	0.394	9385.5 ± 496.7	7405.4 ± 379.0 *	**0.005**
*n*-3	3917.7 ± 167.4	4143.3 ± 233.3	0.447	4470.8 ± 167.5	3853.1 ± 108.1 *	**0.006**
Total	25,149.5 ± 2216.3	28,617.0 ± 2441.6	0.304	22,159.7 ± 1426.4	16,371.1 ± 1134.2 *	**0.005**

SFA: saturated fatty acids; MUFA: monounsaturated fatty acids; PUFA: polyunsaturated fatty acids; *n*-3: omega-3 fatty acids.

## Data Availability

Data are contained within the article and/or available upon request.

## Supplementary Materials

The following supporting information can be downloaded at: https://www.mdpi.com/article/10.3390/biom12040516/s1, Supplemental Figure S1: Growth responses of WT and LepR mutant rainbow trout that were feed deprived for six weeks; Supplemental Figure S2: Liver and muscle glycogen, cortisol, and leptin measurements in WT and LepR mutant rainbow trout that were feed deprived for six weeks; Supplemental Figure S3: Hypothalamic genes involved in feed intake were measured in WT and LepR mutant rainbow trout that were feed deprived for six weeks; Supplemental Table S1: Fatty acid content of liver tissue from WT and LepR mutant rainbow trout that were fed to satiation or fasted for six weeks; Supplemental Table S2: Fatty acid content of adipose tissue from WT and LepR mutant rainbow trout that were fed to satiation or fasted for six weeks.
