# Supplementary material for: Leptin Receptor Deficiency Results in Hyperphagia and Increased Fatty Acid Mobilization during Fasting in Rainbow Trout (Oncorhynchus mykiss)
Source: Biomolecules. 2022 Mar 29;12(4):516. doi: 10.3390/biom12040516 (PMC9028016; doi:10.3390/biom12040516)
Supplement: Supplementary file 1 [file biomolecules-12-00516-s001.zip › biomolecules-1631682-supplementary.pdf]

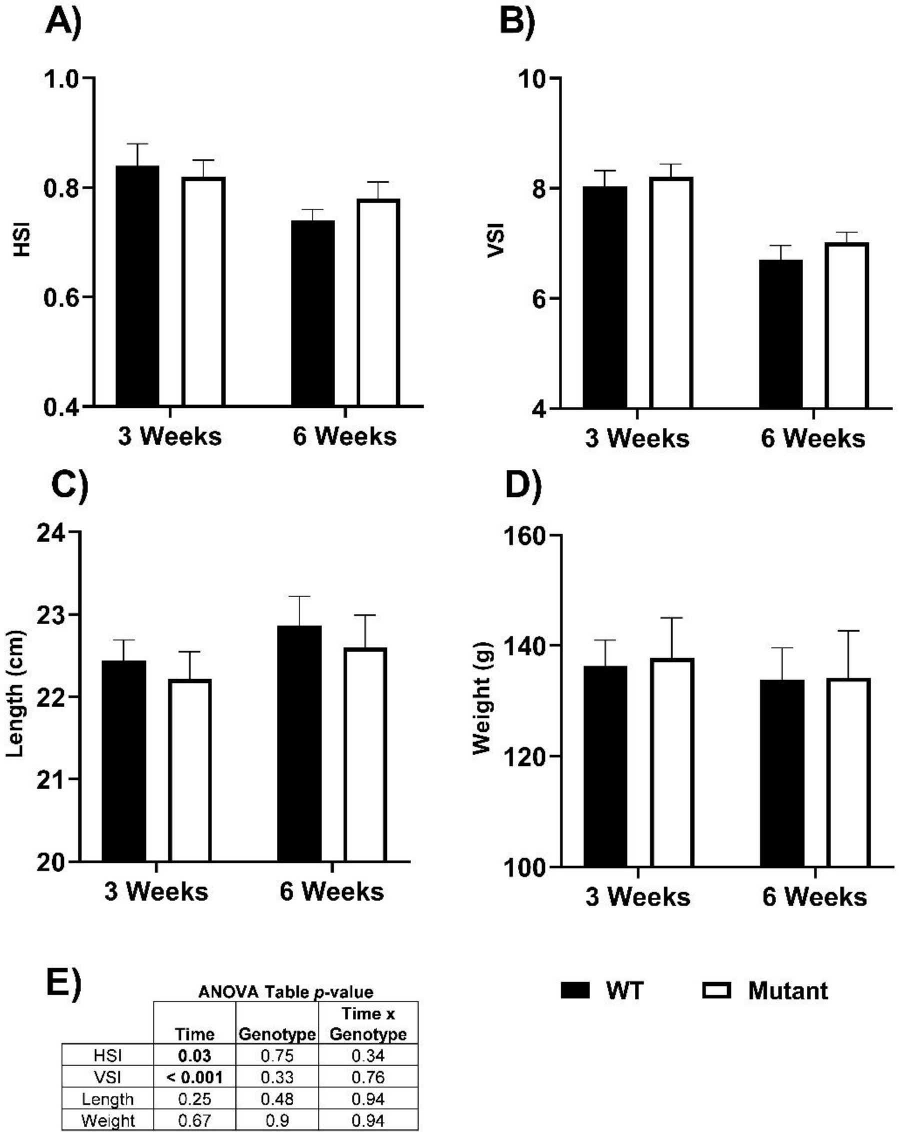

E)

ANOVA Table *p*-value	Time	Genotype	Time x Genotype
HSI	**0.03**	0.75	0.34
VSI	**< 0.001**	0.33	0.76
Length	0.25	0.48	0.94
Weight	0.67	0.9	0.94

Supplemental Figure 1. Growth responses of WT and LepR mutant rainbow trout that were feed deprived for six weeks. **A**) hepatosomatic index (HSI), **B**) viscerosomatic index (VSI), **C**) length (cm), **D**) weight (g), and **E**) ANOVA table. (n = 12-30). The significant *p*-values from different interactions are shown bolded in the ANOVA table in panel **S1E.**

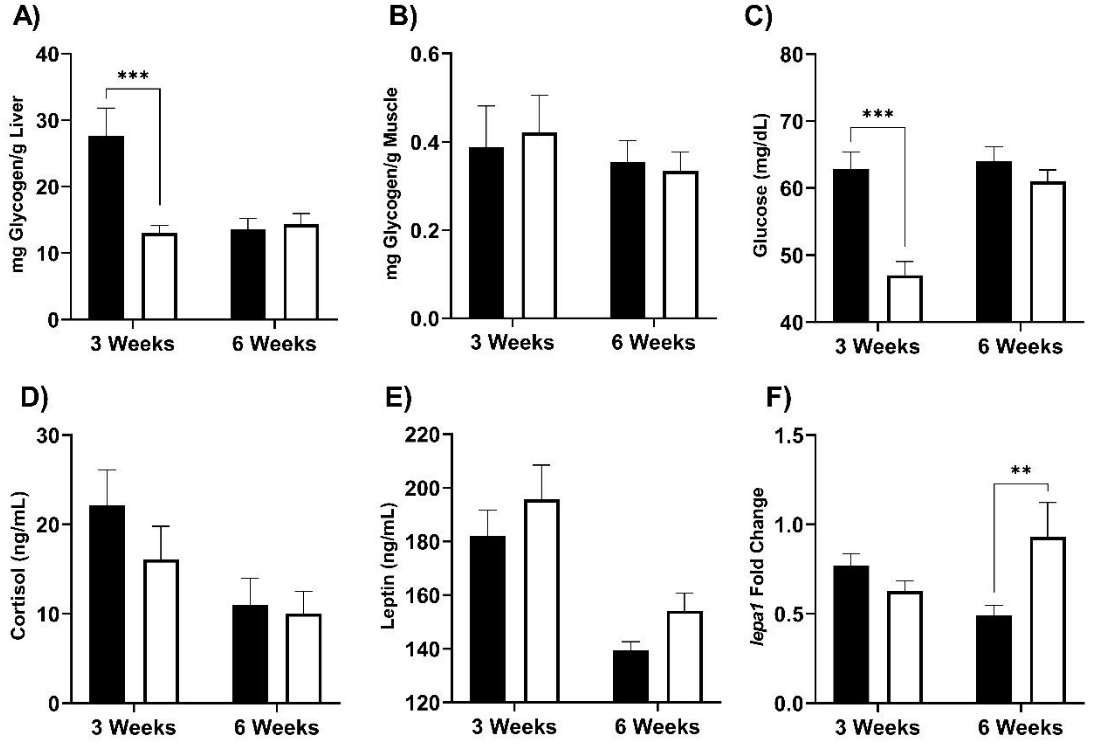

G)

ANOVA Table *p*-value

	Time	Genotype	Time x Genotype
Liver Glycogen	**0.02**	**0.01**	**0.005**
Muscle Glycogen	0.4	0.93	0.71
Cortisol	**0.01**	0.3	0.45
Glucose	**0.001**	**< 0.001**	**0.006**
Leptin	**< 0.001**	0.13	0.96
lepa1	0.91	0.18	**0.01**

Supplemental Figure 2. Liver and muscle glycogen, cortisol, and leptin measurements in WT and LepR mutant rainbow trout that were feed deprived for six weeks. **A**) liver glycogen (mg/g liver), **B**) muscle glycogen (mg/g muscle), **C**) blood glucose (mg/dL), **D**) plasma cortisol (ng/mL), **E**) plasma leptin (ng/mL), **F**) hepatic *lepa1* mRNA expression (fold-change from three-week WT), **G**) ANOVA table. Values reported as means ± SEM. * denotes significant differences between WT and mutant fish within each time point. (**$p = 0.005$, *** $p \leq 0.001$, n = 11-15). The significant *p*-values from different interactions are shown bolded in the ANOVA table in panel **S2G.**

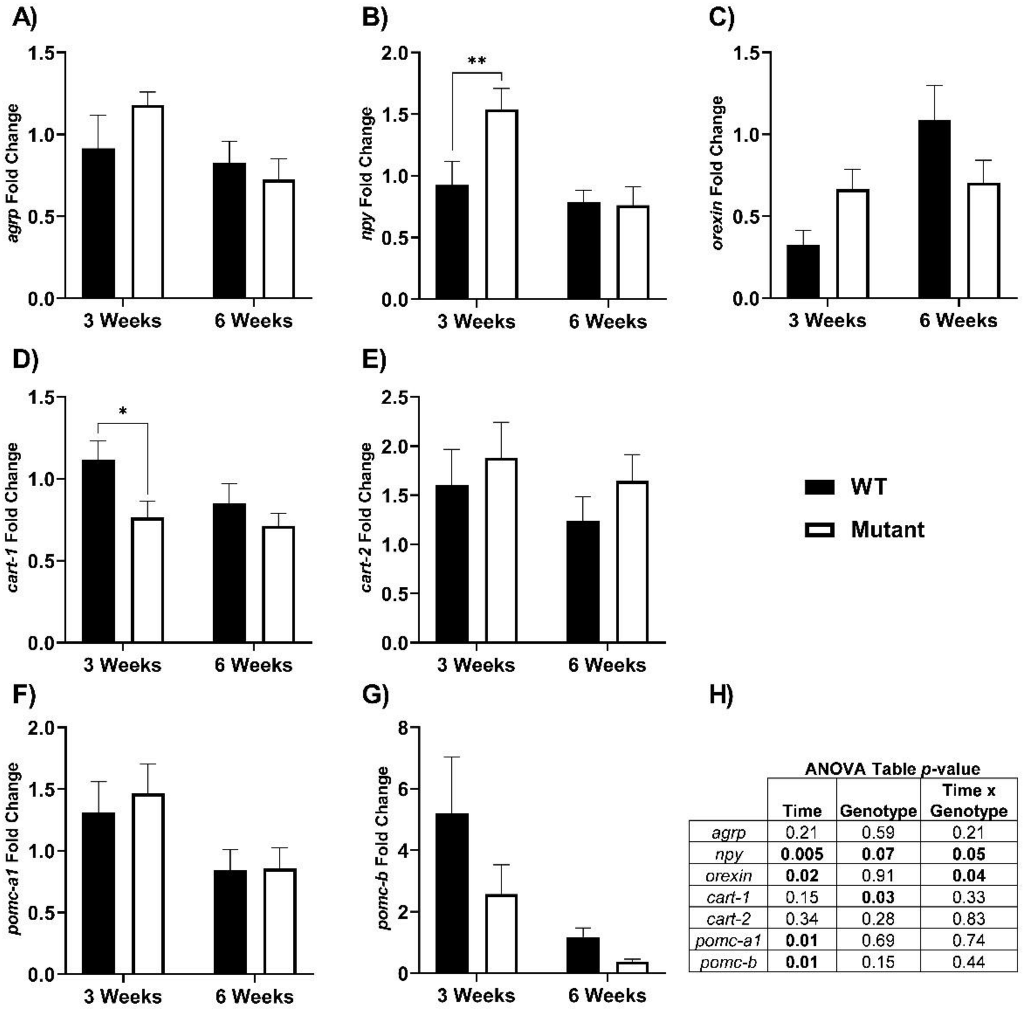

ANOVA Table *p*-value

	Time	Genotype	Time x Genotype
agrp	0.21	0.59	0.21
npy	**0.005**	**0.07**	**0.05**
orexin	**0.02**	0.91	**0.04**
cart-1	0.15	**0.03**	0.33
cart-2	0.34	0.28	0.83
pomc-a1	**0.01**	0.69	0.74
pomc-b	**0.01**	0.15	0.44

Supplemental Figure 3. Hypothalamic genes involved in feed intake were measured in WT and LepR mutant rainbow trout that were feed deprived for six weeks. **A**) *agrp*, **B**) *npy*, **C**) *orexin*, **D**) *cart-1*, **E**) *cart-2*, **F**) *pomc-a1*, **G**) *pomc-b*, **H**) ANOVA table. Values reported as means ± SEM and expressed as fold change from the three-week WT. * denotes significant differences between WT and mutant fish within each time point. (**p* = 0.02, ** *p* = 0.007, n = 8-15). The significant *p*-values from different interactions are shown bolded in the ANOVA table in panel **S3H.**

Supplemental Table 1. Fatty acid content of liver tissue from WT and LepR mutant rainbow trout that were fed to satiation or fasted for six weeks. Data shown is from the final sampling at six-weeks. * and bold values denote a significant difference between WT and mutant fed fish (n = 15).

Liver

**Fatty Acid**	**WT Fed**	**Mutant Fed**	***p*-value**	**WT Fasted**	**Mutant Fasted**	***p*-value**
14:0	283.6 ± 26.6	282.3 ± 18.0	0.969	204.2 ± 13.0	183.2 ± 9.1	0.199
16:0	4511.8 ± 213.7	4682.1 ± 270.1	0.621	6267.0 ± 145.1	6053.6 ± 168.3	0.346
17:0	52.9 ± 2.6	48.0 ± 0.9	0.080	73.2 ± 1.4	74.9 ± 1.2	0.367
18:0	1972.6 ± 91.9	2213.5 ± 112.7	0.104	1801.3 ± 40.6	1792.2 ± 49.2	0.886
20:0	48.6 ± 2.8	54.4 ± 4.4	0.268	27.1 ± 1.1	25.7 ± 1.1	0.381
22:0	51.3 ± 4.5	67.7 ± 6.6*	**0.050**	22.5 ± 1.0	21.5 ± 1.0	0.488
SFA	6985.8 ± 309.7	7410.8 ± 380.6	0.397	8476.8 ± 181.2	8231.6 ± 215.2	0.390
16:1n-7,Z	889.4 ± 117.4	1051.3 ± 168.6	0.435	572.3 ± 26.5	505.7 ± 24.0	0.073
18:1n-9,E	49.1 ± 2.9	53.8 ± 3.2	0.290	50.9 ± 1.4	48.0 ± 1.2	0.124
18:1n-9,Z	4768.4 ± 467.5	6229.3 ± 877.5	0.152	3732.6 ± 134.8	3497.3 ± 148.9	0.245
18:1n-7,Z	602.0 ± 44.4	683.2 ± 50.7	0.234	573.1 ± 16.3	556.8 ± 21.3	0.540
20:1n-9	566.0 ± 42.8	736.2 ± 39.1*	**0.006**	216.6 ± 12.2	211.6 ± 12.6	0.781
24:1n-9	136.9 ± 5.9	149.6 ± 5.0	0.105	169.7 ± 7.0	171.8 ± 12.1	0.873
MUFA	7120.5 ± 648.1	9091.1 ± 1094.4	0.154	5417.7 ± 186.5	5058.6 ± 209.0	0.246
18:2n-6,Z,Z	1909.1 ± 220.1	1755.6 ± 85.9	0.511	3111.6 ± 127.1	2938.6 ± 145.9	0.372
18:3n-6	62.5 ± 5.3	62.5 ± 4.6	0.992	56.1 ± 3.4	54.4 ± 3.5	0.727
18:3n-3	112.3 ± 22.6	88.6 ± 5.2	0.302	257.7 ± 12.0	239.0 ± 12.8	0.294
20:2n-6	447.8 ± 31.4	444.3 ± 19.9	0.923	379.6 ± 13.8	386.1 ± 15.6	0.753
20:3n-6	525.1 ± 29.7	560.4 ± 27.9	0.386	380.4 ± 10.2	402.3 ± 17.3	0.269
20:4n-6	989.2 ± 90.3	1063.8 ± 64.3	0.497	1436.2 ± 28.3	1456.7 ± 35.9	0.886
20:5n-3	384.6 ± 17.0	391.8 ± 19.2	0.779	789.8 ± 27.5	775.5 ± 37.1	0.752
22:4n-6	108.0 ± 8.0	135.3 ± 9.2*	**0.032**	44.1 ± 1.4	42.6 ± 2.3	0.539
22:5n-6	309.9 ± 17.9	373.3 ± 21.7*	**0.032**	143.2 ± 4.2	139.8 ± 5.8	0.656
22:5n-3	175.4 ± 9.8	183.2 ± 10.7	0.588	381.6 ± 13.1	366.0 ± 15.7	0.445
22:6n-3	6185.2 ± 302.5	6436.3 ± 143.2	0.450	8102.1 ± 211.1	8043.2 ± 205.1	0.847
PUFA	11256.3 ± 542.6	11535.5 ± 293.0	0.646	15169.8 ± 309.2	14905.2 ± 366.7	0.581
n-3	6883.9 ± 316.9	7121.1 ± 160.4	0.501	9572.7 ± 237.0	9466.3 ± 233.5	0.757
Total	25362.6 ± 1324.1	27965.4 ± 1536.1	0.213	29064.3 ± 590.4	28222.4 ± 743.3	0.377

SFA: saturated fatty acids; MUFA: monounsaturated fatty acids; PUFA: polyunsaturated fatty acids; n-3: omega-3 fatty acids.

Supplemental Table 2. Fatty acid content of adipose tissue from WT and LepR mutant rainbow trout that were fed to satiation or fasted for six weeks. Data shown is from the final sampling at six-weeks. (n = 15).

Adipose

**Fatty Acid**	**WT Fed**	**Mutant Fed**	***p*-value**	**WT Fasted**	**Mutant Fasted**	***p*-value**
12:0	428.5 ± 12.9	430.2 ± 15.9	0.934	398.6 ± 8.6	389.2 ± 20.2	0.659
14:0	20193.2 ± 459.1	19884.3 ± 443.5	0.632	19278.3 ± 333.7	18486.6 ± 720.4	0.307
15:0	1540.0 ± 27.7	1492.8 ± 35.2	0.306	1447.0 ± 24.5	1411.4 ± 51.1	0.518
16:0	166095.2 ± 2869.9	166577.4 ± 3091.6	0.910	153710.9 ± 2444.9	145010.4 ± 6336.7	0.189
17:0	1672.2 ± 41.3	1640.8 ± 39.0	0.585	1590.2 ± 31.2	1563.2 ± 53.1	0.655
18:0	47898.0 ± 767.3	49979.1 ± 1231.5	0.170	49926.9 ± 857.9	47691.9 ± 1683.6	0.229
20:0	1921.8 ± 38.6	1945.5 ± 43.4	0.689	2092.5 ± 17.6	2055.6 ± 62.6	0.551
22:0	1470.3 ± 29.1	1501.5 ± 28.7	0.453	1448.2 ± 17.3	1423.8 ± 48.1	0.619
24:0	572.9 ± 12.6	554.7 ± 12.5	0.313	558.1 ± 9.9	564.2 ± 19.6	0.774
SFA	241933.8 ± 3675.8	244187.7 ± 4442.3	0.701	230637.2 ± 2941.3	218769.2 ± 8704.7	0.183
14:1n-5,Z	539.7 ± 17.4	536.8 ± 14.4	0.901	504.0 ± 16.5	476.6 ± 22.7	0.328
16:1n-7,Z	57430.5 ± 1575.4	58264.7 ± 1433.1	0.698	53006.6 ± 1655.4	49767.3 ± 2302.2	0.255
18:1n-9,Z	291364.6 ± 4530.6	303370.7 ± 5029.8	0.089	304867.1 ± 4331.7	290716.8 ± 9516.9	0.169
18:1n-7,Z	32251.5 ± 452.8	32145.8 ± 468.2	0.873	30777.3 ± 434.2	29851 ± 1115.9	0.423
20:1n-9	20079.2 ± 588.7	20073.7 ± 446.3	0.994	22329.9 ± 518.9	22744.2 ± 981.1	0.701
22:1n-9	3041.5 ± 59.4	3057.2 ± 45.5	0.834	3612.3 ± 60.5	3546.5 ± 125.3	0.626
24:1n-9	1967 ± 50.0	1945.4 ± 41.8	0.741	2241.0 ± 64.6	2176.4 ± 74.3	0.516
MUFA	411384.5 ± 6296.3	424036.6 ± 6681.0	0.181	422067.6 ± 5816.8	403801.8 ± 13638.6	0.207
18:2n-6,Z,Z	227144.7 ± 3590.6	221560.6 ± 4003.5	0.311	228963.5 ± 3561.7	221055.9 ± 5521.2	0.228
18:3n-6	3945.3 ± 168.0	3991.7 ± 110.6	0.817	3731.6 ± 87.5	3658.6 ± 118.7	0.619
18:3n-3	21630.7 ± 449.2	21111.0 ± 453.9	0.424	19340.0 ± 288.9	18836.9 ± 768.5	0.524
20:2n-6	14233.0 ± 229.9	13840.3 ± 264.9	0.276	14115.4 ± 322.2	14052.5 ± 489.8	0.913
20:3n-6	6674.5 ± 207.6	6502.2 ± 160.6	0.512	6783.0 ± 211.7	6678.0 ± 277.9	0.763
20:3n-3	1819.3 ± 46.1	1715.1 ± 32.7	0.073	1723.0 ± 33.9	1619.1 ± 68.3	0.667
20:4n-6	5454.3 ± 219.1	5810.7 ± 106.2	0.196	5437.9 ± 131.9	5314.1 ± 208.5	0.611
22:2n-6	1556.8 ± 35.1	1518.9 ± 34.9	0.452	1600.7 ± 34.7	1600.3 ± 57.2	0.995
20:5n-3	10907.2 ± 239.8	10968.4 ± 246.9	0.860	10279.5 ± 219.7	9854.0 ± 467.4	0.397
22:4n-6	831.6 ± 37.1	880.9 ± 35.1	0.343	920.4 ± 26.3	877.0 ± 45.3	0.400
22:5n-6	1178.9 ± 74.7	1323.2 ± 70.5	0.171	1285.1 ± 44.2	1258.0 ± 66.1	0.730
22:5n-3	3885.1 ± 145.9	3789.3 ± 122.5	0.617	4380.3 ± 144.6	4188.6 ± 193.0	0.427
22:6n-3	33654.7 ± 806.6	34446.1 ± 954.7	0.535	35921.6 ± 760.2	35431.7 ± 8612.9	0.782
PUFA	333073.7 ± 4103.9	327629.6 ± 5010.7	0.412	334655.2 ± 4475.8	324637.4 ± 8612.9	0.293
n-3	71897.0 ± 1069.3	72029.9 ± 1323.0	0.939	71644.2 ± 1142.2	70002.2 ± 2879.0	0.582
Total	986392.0 ± 11800.2	995853.8 ± 13878.0	0.610	987360.0 ± 9163.9	947208.3 ± 30157.3	0.188

SFA: saturated fatty acids; MUFA: monounsaturated fatty acids; PUFA: polyunsaturated fatty acids; n-3: omega-3 fatty acids.
